# Toosendanin inhibits adipogenesis by activating Wnt/β-catenin signaling

**DOI:** 10.1038/s41598-018-22873-x

**Published:** 2018-03-15

**Authors:** Tian-xing Chen, Xiao-ying Cheng, Yun Wang, Wu Yin

**Affiliations:** 1 The State Key Lab of Pharmaceutical Biotechnology, College of life Sciences, Nanjing University, Nanjing, 210046 China; 2 Jiangsu Key Laboratory for Pharmacology and Safety Evaluation of Chinese Materia Medica, School of pharmacy, Nanjing University of Chinese Medicine, Nanjing, 210023 China; 3 Nanjing KMK Pharmaceutical Co., Nanjing, 210024 China

## Abstract

Toosendanin (TSN), a triterpenoid extracted from Melia toosendan, has been reported to possess anti-oxidant, anti-inflammatory, anti-allergic, and anti-arthritic activities. However, its anti-adipogenic effect remains unknown. Here, we found that TSN dose-dependently attenuated lipid accumulation in preadipocytes 3T3-L1 as evidenced by Oil Red O staining. TSN also significantly downregulated mRNA and protein levels of adipocytokines (adiponectin and leptin), CCAAT/enhancer binding proteins α (C/EBP-α), peroxisome proliferator-activated receptor γ (PPAR-γ), fatty acid synthase, and acetyl-CoA carboxylase in adipocytes. To understand the mechanism, we observed that TSN effectively activated Wnt/β-catenin pathway, in which TSN increased low density lipoprotein receptor related protein 6, disheveled 2, β-catenin, and cyclin D1 expression levels, while it inactivated glycogen synthase kinase 3β by enhancing its phosphorylation. Moreover, TSN reduced weight of gonadal white fat and serum triacylglycerol (TAG) content in high-fat diet (HFD)-fed mice. Interestingly, the *in vivo* studies also demonstrated that TSN promoted the expression of β-catenin, but accordingly repressed C/EBP-α and PPAR-γ expression in HFD-induced mice. Overall, TSN is capable of inhibiting the lipogenesis of adipocytes by activating the Wnt/β-catenin pathway, suggesting potential application of TSN as a natural anti-obesity agent.

## Introduction

Obesity promotes the prevalence of a variety of diseases including type 2 diabetes, hyperlipidemia, hypertension, cardiac injury, and cancer^1,2^, which heavily threatens public health. Obesity is originated from the excessive lipid accumulation of adipose tissue associated with adipogenesis. The adipogenesis is a complex process involving preadipocyte proliferation, differentiation, intracellular lipid accumulation, and changes in genes expression^3^. Among these processes, adipocyte differentiation is tightly regulated by lots of key adipogenic transcription factors, including nuclear receptor peroxisome proliferator activated receptor gamma (PPAR-γ) and members of the CCAAT/enhancer binding protein (C/EBP) family^4,5^. These transcription factors are indispensable for expression of adipocyte-specific genes, such as adipocyte fatty acid binding protein (aP2), lipoprotein lipase (LPL), fatty acid synthase (FAS), acetyl-CoA carboxylase (ACC), leptin, and adiponectin^6^.

Increasing evidences suggest that the wingless-type MMTV integration site (Wnt)/β-catenin serves as a negative regulator for adipocyte differentiation^7^. In the canonical Wnt/β-catenin signaling, Wnt ligands bind to frizzled receptors and low density lipoprotein receptor-related protein 5/6 (LRP5/6) co-receptors. Following this interaction, disheveled (DVL) gets phosphorylated and the glycogen synthase kinase 3 (GSK3)-AXIN-adenomatous polyposis coli (APC) complex is disrupted, which promotes the stabilization and nuclear translocation of β-catenin^7^. In the nucleus, β-catenin positively regulates its target gene cyclin D1 (CCND1), which downregulates major adipogenic transcription factors, such as PPAR-γ and C/EBP-α^7–9^. Therefore, activating β-catenin pathway and its components could be considered as effective strategies to combat obesity.

Natural products that modulate expression of proteins involved in adipogenesis have attracted much attention. For example, pterostilbene inhibits adipocyte differentiation by targeting C/EBP homologous protein-10 (CHOP-10) in 3T3-L1^10^; allopurinol, quercetin, and rutin ameliorate lipid accumulation in fructose-fed rats^11^; thiacremonone attenuates lipid accumulation partially mediated via AMPK activation in 3T3-L1 adipocytes^12^. The fruit of *MeliatoosendanSieb et Zucc*, also known as Chuan Lian-Zi in Chinese medicine, has significant analgesic effects^13^; in addition, it can be used to kill parasites such as roundworms^14^. Toosendanin (TSN) is the main active component of the fruit of *MeliatoosendanSieb et Zucc*; it can selectively block acetylcholine (Ach) release and has antibotulismic effects both *in vivo* and *in vitro*^15–17^. In recent years, TSN has been demonstrated to induce apoptosis on a variety of cancer cells^18^. We previously described that TSN, when used at low concentrations, was able to reverse lung cancer cells resistance toward TRAIL-mediated apoptosis by upregulating TRAIL receptors^19^. All of the data suggest potential application of TSN in cancer treatment.

Interestingly, the fruit of *MeliatoosendanSieb et Zucc* is also an important ingredient in some Chinese prescriptions to treat fat liver diseases, suggesting it may influence lipid metabolism. However, the anti-adipogenic effect of TSN has not yet been investigated. To fill this gap and test the hypothesis, we performed this study, and found that TSN can significantly attenuate adipogenesis through activating the Wnt/β-catenin signaling.

## Materials and Methods

### Materials

**Figure 1:**
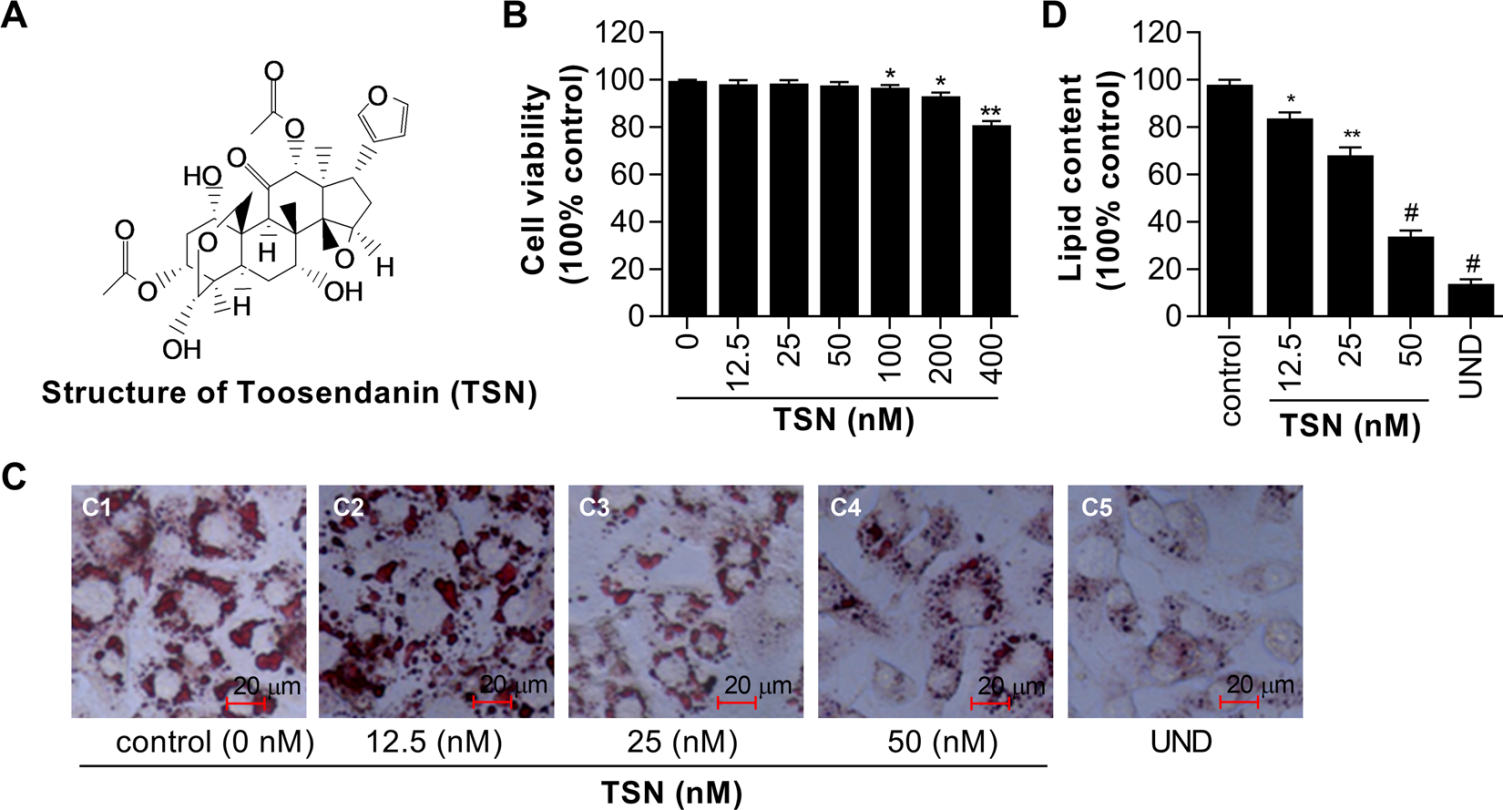
TSN inhibits lipid accumulation in 3T3-L1 adipocytes. Preadipocytes (3T3-L1 cells) were induced to differentiate with increasing concentrations (nM) of TSN for 6 days. The assays were performed on the 6th day. (**A**) Structure of TSN. (**B**) Cell viability was determined by MTT assay. Values are presented as mean ± SD of data from three independent experiments. *P < 0.05, **P < 0.01 vs. control (0 nM TSN). (**C**) The cellular lipid content was assessed by Oil Red O staining. (**D**) Quantification by extracting Oil Red O-stained lipid droplets with 100% isopropanol and OD (540 nm) was measured with a M200 PRO multi-functional microplate reader. Values are mean ± SD of data from three separate experiments. UND: undifferentiated samples; *P < 0.05, **P < 0.01 and ^#^*P* < 0.001 vs. control (0 nM TSN).

The chemical structure of TSN is shown in Fig. 1A. TSN was provided by National Institutes for Food and Drug Control and the purity is higher than 98.5%. Dulbecco’s modified eagle’s medium (DMEM) and fetal bovine serum (FBS) were purchased from Hyclone (Utah, USA). Bovine calf serum (BCS) was purchased from Gibco (New York, USA). 3T3-L1 preadipocyte was purchased from American Type Culture Collection (ATCC, Virginia, USA). Insulin, 3-isobutyl-1-methylxanthine (IBMX), dexamethasone (DEX), Oil Red O, rosiglitazone, 3-[4,5-dimethylthiazol-2-yl]-2,5-diphenyltetrazolium bromide (MTT), and protease inhibitor cocktail were purchased from Sigma-Aldrich (Missouri, USA). The triglyceride (GPO-Trinder) kit was purchased from Nanjing Jiancheng Bioengineering Institute (Nanjing, China). The ELISA kit for detecting the index of ALT and AST in liver was purchased from Nanjing Jiancheng Bioengineering Institute (Nanjing, China). Reverse transcription premix was purchased from TOYOBO (Osaka, Japan). Both TRIZOL reagent and the SYBR green PCR system were from Invitrogen (California, USA). Total protein extraction kit for adipose tissues/cultured adipocytes was from Invent Biotechnologies (Eden Prairie, USA). RIPA lysis buffer and Bradford protein assay kit were purchased from Beyotime (Shanghai, China). Polyvinylidene fluoride membrane was purchased from Merck Millipore (Massachusetts, USA). Antibodies against phosphorylated GSK3β, PPAR-γ, and α-tubulin were purchased from Cell Signaling Technology (Massachusetts, USA). The antibodies against ERK, p-ERK (Thr202/Tyr204), p38, p38 (Thr202/Tyr204), JNK, p-JNK (Thr183/185) were from Cell Signal Technology. Antibody against β-catenin and other secondary antibodies (goat anti-mouse or goat anti-rabbit horseradish peroxidase-conjugated IgG) (1:50000 dilution) were all purchased from Bioworld (Minnesota, USA). β-catenin small interfering RNA (siRNA) and control siRNA were purchased from Genepharma (Shanghai, China). Lipofectamine^TM^ 2000 transfection reagent was purchased from Invitrogen (California, USA).

### Cell culture and differentiation

The 3T3-L1 preadipocytes were differentiated as previously described^12^. 3T3-L1 preadipocytes were cultured in DMEM (10% FBS). To induce differentiation, two days post-confluence (day 0) cells were incubated with induction medium supplemented with MDI (5 μg/mL insulin, 1 μM dexamethasone, 0.5 mM methylisobutylxanthine) and 10% FBS for 48 h. The medium was then replaced with DMEM containing 5 μg/mL insulin and 10% FBS for another 48 h (day 2). On 4^th^ day, the cells were maintained in DMEM/10% FBS until most of the cells were differentiated into mature adipocytes with abundant lipid droplets at 6^th^ day. TSN was added to the culture medium at different concentrations during the adipogenic period (6 days). Cells cultured in DMEM with 10% FBS but without induction medium were regarded as the undifferentiated group (UND).

### MTT cell viability assay

The cell viability was determined by MTT assay in 96-well plates^12^. Preadipocytes were seeded and induced to differentiate in induction medium with increasing concentrations of TSN for 6 days. On 6th day, the treatment medium was removed and replaced with 100 μL 10% FBS fresh medium and 20 μL MTT cell viability reagents. Then, cells were incubated at 37 °C in a 5% CO_2_ atmosphere for 1 h. The absorbance at 490 nm was measured on a M200 PRO multifunctional microplate reader (TECAN, Switzerland).

### Oil red O staining and quantification of lipid content

The cellular lipid content was evidenced by Oil Red O staining as previously described^12^. At 6th day, cells were washed and fixed in 4% paraformaldehyde for 30 min, stained with Oil Red O working solution and incubated for another 20 min at room temperature. After washed three times with PBS, cells were photographed by a light microscope (Olympus, Japan). Next, quantitation was carried out by extracting Oil Red O-stained lipid droplets with 100% isopropanol and the OD at 540 nm was measured with a M200 PRO multifunctional microplate reader (TECAN, Switzerland). Finally, lipid accumulation for each TSN-administrated group is expressed relative to that of MDI-differentiated cells.

### Reverse transcription-polymerase chain reaction (RT-PCR)

The reverse transcription-polymerase chain reaction (RT-PCR) and the quantitative real-time PCR (Q-PCR) were performed as previously described^20^. Total RNA was extracted from 3T3-L1 adipocytes and gonadal fat tissue using TRIZOL reagent. The cDNA was obtained by reverse transcription in a 20 μL reaction containing 2 μg of total RNA, oligo (dT), and reverse transcription premix. Next, normal amplification consisted of 28–30 cycles as follows: denaturing at 94 °C for 30 s, annealing at 56 °C for 1 min and extending at 72 °C for 30 s, followed by a final 10 min incubation at 72 °C. The PCR was performed using a Bio-Rad T100^TM^ Thermal Cycler (Bio-Rad, Hercules, USA), PCR products were electrophoresed by 2% agarose gel electrophoresis and visualized using a Tanon 2000 imaging system (Tanon, Changxing, China).

The Quantitative real-time PCR (Q-PCR) reactions were performed with the SYBR green PCR system in an ABI 7500 thermal cycler. The cycling conditions were as follows: 95 °C for 3 min; followed by 40 cycles involving denaturing at 95 °C for 10 s, annealing at 60 °C for 5 s and extension at 72 °C for 10 s. Expression of mRNAs was normalized by the mRNA levels of β-actin, which was used as an internal control. The primers were shown as follows: C/EBP-α: sense, 5′-GCGCAAGAGCCGAGATAAAG-3′, antisense, 5′-CGGTCATTGTCACTGGTCAACT-3′; C/EBP-β: sense, 5′-CGCAGACAGTGGTGAGCTT-3′, antisense, 5′-CTTCTGCTGCATCTCCTGGT-3′; C/EBP-δ: sense, 5′-CAAGCTGAGCGACGAGTACA-3′, antisense, 5′-AGCTGCTCCACCTTCTTCTG-3′; PPAR-γ: sense, 5′-TGAACGTGAAGCCCATCGAG-3′, antisense, 5′-CTTGGCGAACAGCTGAGAGG-3′; FAS: sense, 5′-ATCCTGGAACGAGAACACGATCT-3′, antisense, 5′-AGAGACGTGTCACTCCTGGACTT-3′; ACC: sense, 5′-AGCTGATCCTGCGAACCT-3′, antisense, 5′-GCCAAGCGGATGTAAACT-3′; Adiponectin: sense, 5′-GGCAGGCATCCCAGGACATC-3′, antisense, 5′-TCTCACCCTTAGGACCAAGAAGAC-3′; Leptin: sense, 5′-GAGACCCCTGTGTCGGTTC-3′, antisense, 5′-CTGCGTGTGTGAAATGTCATTG-3′; LRP6: sense, ACCTCAATGCGATTTGTTCC, antisense, GGTGTCAAAGAAGCCTCTGC; DVL2: sense, GCTTCCACATGGCCATGGGC, antisense, CACTGCTGGTGAGAGTCACAG; β-Catenin: sense, GCCAAGTGGGTGGTATAGAG, antisense, CTGGGTATCCTGATGTGC; CCND1: sense, AAATCGTGGCCACCTGGAT, antisense, CATCCGCCTCTGGCATTTTG; β-Actin: sense, AGAACATCATCCATGCATCCA, antisense, GCCTGCTTCACCACCTTCTTG.

### Protein extraction and immunoblotting

The western blot was performed as previously described^20^. 3T3-L1 adipocytes were lysed using RIPA lysis buffer with a protease inhibitor cocktail. Gonadal fat tissue was lysed by the total protein extraction kit for adipose tissues from Invent Biotechnologies (Eden Prairie, USA). The protein concentrations of lysate were determined by using the Bradford protein assay kit. Equal amounts of protein (30 to 60 μg) in each sample were separated by 10% SDS-polyacrylamide gel electrophoresis (SDS-PAGE) and transferred to polyvinylidene fluoride membranes. The membranes were blocked with 5% skimmed milk in Tris-buffered saline containing 0.1% Tween-20 for 1 h at room temperature and then incubated overnight with primary antibodies against C/EBP-α, PPAR-γ, FAS, ACC, adiponectin, leptin, β-catenin, phosphorylated GSK3β, and α-tubulin (1:1000 dilution) at 4 °C. After washing three times in Tris-buffered saline containing 0.1% Tween-20, the membranes were incubated with goat anti-mouse or goat anti-rabbit horseradish peroxidase-conjugated IgG secondary antibody. Target proteins were detected with the enhanced chemiluminescence (ECL) detection system and visualized with the Tanon imaging system and the gene snap program (Tanon, Changxing, China).

### Knockdown of β-catenin by siRNA transfection

β-catenin knockdown by siRNA transfection was performed as described in instruction of lipofectamine^TM^ 2000 transfection reagent. Two days after confluence, 3T3-L1 cells were cultured in serum-free medium for 1 h and transfected with 60 nM of β-catenin siRNA or control siRNA using the transfection reagent. After 9 h, the transfected cells were differentiated as described above.

### Luciferase reporter assay

The 3T3-L1 adipocytes in 24-well plates were transfected with empty vector (pGL3 basic) or the β-catenin promoter (−220 bp to +10 bp) plasmid, which was cloned into pGL3 basic. After transfection for 10 h, the cells were incubated for another 10 h. Then, luciferase activity was determined using a Dual Luciferase^®^ Reporter Assay kit (Promega, Wiscosin, USA) following the manufacturer’s instructions.

### Hematoxylin and eosin (H & E) staining

The H & E staining was performed as previously described^21^. The gonadal fat tissues and liver from mice were fixed in 4% formalin buffer and the fixed specimens were processed to paraffin blocks, sectioned, and subject to hematoxylin-eosin (H & E) staining for histological analysis according to the standard protocols.

### Animal experiment

Eight weeks old male C57 BL/C6 mice used in this study were approved by the Animal Care and Protection Committee of Nanjing University-Gulou hospital (SYXK 2004-0013). The authors confirmed that all animals received human care and all animal experiments were performed in accordance with the relevant guidelines and regulations. This experiment was conducted as previously described^22^. The mice were fed with a 45% high fat diet (HFD) from FBSH (Shanghai, USA) for one month and a half, the mice were grouped randomly into NFD + placebo, HFD + placebo, HFD + TSN (0.1 mg/kg·d) and 8 mice for each group. The effect of TSN on the levels of ALT and AST (U/L) in liver was examined by ELISA kit according to the manufacturer instruction. Body weight, food intake, and weight of gonadal white fat (gWAT) were calculated. The triacylglycerol (TAG) content in serum and gonadal fat tissue was determined in duplicate using the triglyceride (GPO-Trinder) kit. The gonadal fat tissues were subjected to hematoxylin-eosin (H & E) staining for histological analysis as described above. The mRNA expressions of C/EBP-α, PPAR-γ, and β-catenin were examined by real-time PCR. Total protein of gonadal white fat was extracted by total protein extraction kit for adipose tissues from Invent Biotechnologies, and the protein samples were subjected to western blot analysis.

### Statistics

The values are presented as means ± SD. Statistical differences between two means were determined using Student’s t-test (unpaired, two-tailed) using Graphpad prism 5 to calculate p-values (*p < 0.05, **p < 0.01, ^#^p < 0.001 and ^Ф^p < 0.001). P < 0.05 was considered statistically significant between groups.

## Results

### Effect of TSN on lipid accumulation of adipocytes

The 3T3-L1 preadipocytes were treated with TSN at various concentrations (12.5, 25, 50, 100, 200, and 400 nM) for 6 days. Cell viability was determined by MTT assay after treatment. As shown in Fig. 1B, cell viability was decreased by TSN at the concentration of 100 nM, 200 nM, and 400 nM while not affected by TSN at the concentration of 12.5 nM, 25 nM, and 50 nM. Therefore, the concentrations ranging from 12.5 nM to 50 nM are appropriate for the treatment of cells in the subsequent experiments. The effect of TSN on lipid accumulation was examined by Oil Red O staining (Fig. 1C). Compared with the control cells that were incubated with induction medium (MDI) only, TSN reduced cellular lipid droplets in a dose dependent manner and the most effective dosage for inhibition was TSN at 50 nM. To quantify the content of lipids, the OD_540_ value of cells after induction and drug treatments were examined. TSN, when used at 12.5 nM, 25 nM, and 50 nM, significantly decreased the cellular lipid contents by 15.93% (P < 0.05), 31.24% (P < 0.01), and 65.5% (P < 0.001) respectively, as compared with the control cells (Fig. 1D).

### TSN downregulates the expression of transcription factors

**Figure 2:**
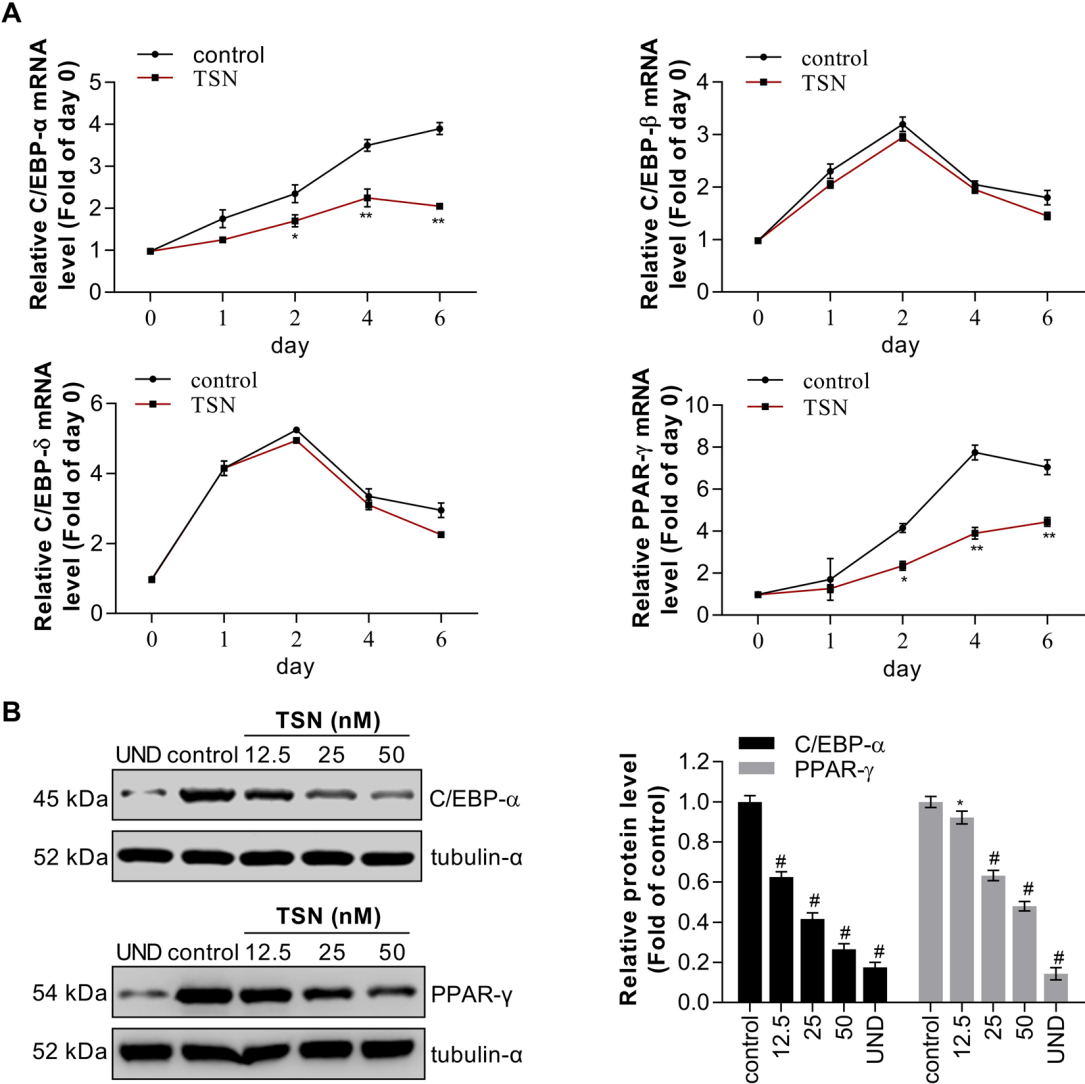
TSN downregulates expression of transcription factors. (**A**) Preadipocytes were induced to differentiate with 50 nM TSN and harvested at days (0, 1, 2, 4 and 6) during the differentiation period. The mRNA expression of C/EBP-α, C/EBP-β, C/EBP-δ and PPAR-γ was analyzed by real-time RT-PCR. Values are presented as mean ± SD of data from three separate experiments. *P < 0.05, **P < 0.01 vs. control (0 nM TSN). (**B**) Preadipocytes were induced to differentiate with increasing concentrations (12.5, 25 and 50 nM) of TSN for 6 days. At 6th day, the protein levels of C/EBP-α and PPAR-γ was detected by western blot, tubulin-α as loading control and corresponding semi-quantitative analysis was based on optical density with image j software. Values are presented as mean ± SD of data from three separate experiments. UND: undifferentiated samples; **P < 0.01 and ^#^*P* < 0.001 vs. control (0 nM TSN).

Transcriptional factors such as C/EBP-α, C/EBP-β, C/EBP-δ, and PPAR-γ play critical roles in the process of adipogenesis. Thus, we examined the effect of TSN on the mRNA levels as well as protein levels of these transcription factors. Preadipocytes were treated with differentiate-inducing medium along with 50 nM TSN and harvested at indicated time. Real time PCR was performed to analyze the mRNA expressions of C/EBP-α, C/EBP-β, C/EBP-δ, and PPAR-γ. The changes of gene expression during differentiation were shown in Fig. 2A. After treatment with 50 nM TSN for a successive 6 days, the mRNA levels of C/EBP-α and PPAR-γ were all decreased. At the 6^th^ day, the mRNA levels of C/EBP-α and PPAR-γ in TSN-treated cells was nearly 53% and 62% of the control cells. Notably, TSN also demonstrated a weak inhibitory effect on the mRNA expressions of C/EBP-β and C/EBP-δ, the upstream regulators of PPAR-γ and C/EBP-α, but the effect was not significant (P > 0.05). Consistent with the results obtained by real-time PCR, the protein levels of C/EBP-α and PPAR-γ were also reduced by 0.73-fold and 0.52-fold (also as 73% and 52%) in TSN (50 nM)-treated cells, as compared with control cells (Fig. 2B). Meanwhile, the inhibitory effect of TSN on C/EBP-α and PPAR-γ protein expressions was dose dependent.

### TSN suppresses the expressions of lipogenic enzymes and adipocytokines

**Figure 3:**
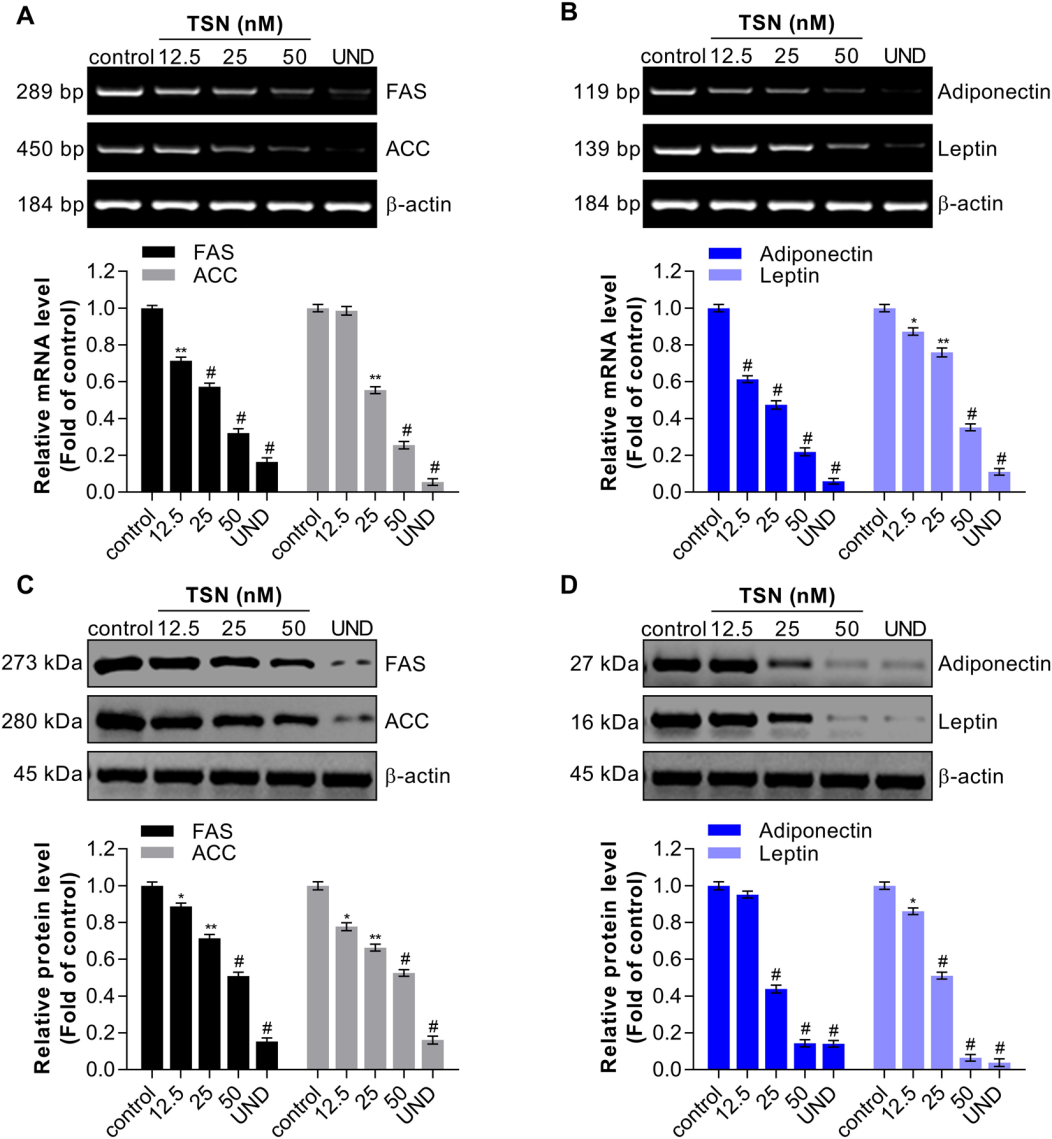
TSN negatively regulates the expression of lipogenic enzymes and adipocytokine in 3T3-L1 adipocytes. (**A**) Effects of TSN treatment with increasing concentrations (nM) on the mRNA expression of lipogenic enzymes, such as FAS and ACC, detected by RT-PCR. The corresponding semi-quantitative analysis was practiced according to optical density with image j software. The results are expressed as mean ± SD (Fold of control) of three independent experiments. UND: undifferentiated samples; *P < 0.05, **P < 0.01 and ^#^*P* < 0.001 vs. control (0 nM TSN). (**B**) Effects of TSN treatment on the mRNA expression of adipocytokines, such as adiponectin and leptin, analyzed by RT-PCR. β-actin was used as an internal control. The results are expressed as mean ± SD (fold of control) of three independent experiments. *P < 0.05, **P < 0.01 and ^#^*P* < 0.001 vs. control (0 nM TSN). (**C**) and (**D**) Effects of TSN treatment on the protein levels of FAS, ACC and adiponectin, leptin, analyzed by western blot and β-actin was used as an internal control. The results are expressed as mean ± SD (fold of control) of three independent experiments. UND: undifferentiated samples; *P < 0.05, **P < 0.01 and ^#^*P* < 0.001 vs. control (0 nM TSN).

Besides transcriptional factors as shown above, TSN also decreased the mRNA expressions of some key lipogenic enzymes, including FAS and ACC, in a dose-dependent manner (Fig. 3A). After 50 nM TSN incubation, the levels of FAS and ACC mRNA expression were significantly inhibited by 0.68-fold (68%) and 0.75-fold (75%), respectively, as compared to the control cells (differentiation but no TSN). Following 50 nM TSN treatment, the levels of adiponectin and leptin mRNA expression in 3T3-L1 differentiated adipocytes were markedly decreased by 0.79-fold (79%) and 0.67-fold (67%), respectively, as compared to the control (Fig. 3B). On the other hand, the protein levels of FAS and ACC, adiponectin and leptin protein expression were repressed by 50 nM TSN, as shown in Fig. 3C,D. Therefore, TSN can effectively inhibit the expressions of lipogenic enzymes and adipocytokine genes during 3T3-L1 adipocyte differentiation.

### TSN activates the expression of the Wnt/β-catenin signaling components

**Figure 4:**
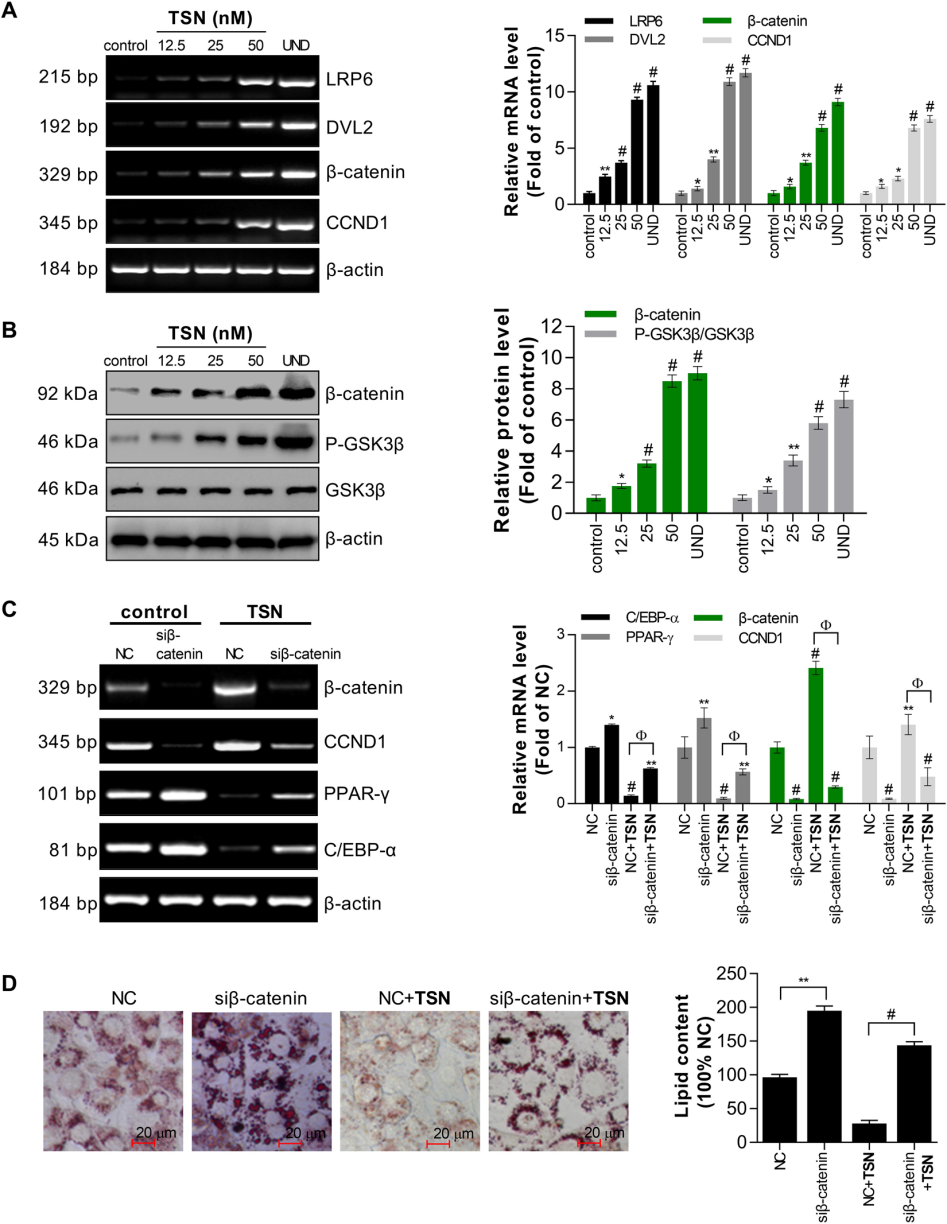
TSN activates the Wnt/β-catenin pathway in 3T3-L1 adipocytes. (**A**) Effects of TSN treatment on the mRNA expression of LRP6, DVL2, β-catenin, and CCND1 analyzed by RT-PCR; the differentiated group without TSN treatment served as the control group. Additionally, the corresponding semi-quantitative analysis was based on optical density determined with image J software. β-actin was used as an internal control. Values are expressed as mean ± SD (fold of control) of three independent experiments. UND: undifferentiated samples; *P < 0.05, **P < 0.01 and ^#^*P* < 0.001 vs. control (0 nM TSN). (**B**) Effects of TSN treatment on the protein expression of β-catenin and p-GSK3β analysed by western blot. β-actin was used as an internal control. The results are expressed as mean ± SD (fold of NC or control) of three independent experiments. UND: undifferentiated samples; *P < 0.05, **P < 0.01 and ^#^*P* < 0.001 vs. control. (**C**) Effects of β-catenin siRNA on the mRNA expression of β-catenin, CCND1, PPAR-γ, and C/EBP-α in the presence and absence of TSN determined by RT-PCR, β-actin was used as an internal control. Values are expressed as mean ± SD (fold of NC or control) of three independent experiments. *P < 0.05, **P < 0.01 and ^#^*P* < 0.001 vs. control; ^Ф^*P* < 0.001 vs. NC + TSN. (**D**) Increase of lipid content after β-catenin siRNA transfection was evidenced by Oil Red O staining and determined at OD_540 nm_ with a M200 PRO multi-functional microplate reader. Scale bars: 20 μM. Values are expressed as mean ± SD (fold of NC or control) of three independent experiments. **P < 0.01 vs NC and ^#^*P* < 0.001 vs. NC + TSN.

It has been reported that preadipocytes differentiation may be suppressed by activated Wnt/β-catenin signaling^7^. In order to examine whether the inhibitory effect of TSN on adipocyte differentiation by Wnt/β-catenin signaling, we performed RT-PCR analysis and found that TSN upregulated the mRNA expression of genes in the Wnt/β-catenin signaling including LRP6, DVL2, β-catenin, and CCND1, as showed in Fig. 4A. Meanwhile, we found that TSN may activate β-catenin promoter and suppressed β-catenin degradation, which was enhanced by cycloheximide (CHX) (Supporting Fig. 1A,B). Furthermore, TSN treatment efficiently activated β-catenin protein levels but inactivated GSK3β by enhancing its phosphorylation (Fig. 4B). Therefore, TSN may activate Wnt/β-catenin signaling to inhibit adipogenesis. To test this presumption, β-catenin siRNA was transfected into 3T3-L1 adipocytes with or without TSN treatment. The data showed that the mRNA expression of β-catenin and CCND1 were effectively decreased by the β-catenin siRNA, as compared with cells transfected with control siRNA in presence or absence of TSN. TSN-induced downregulation of mRNA expression of the adipogenic transcription factors PPAR-γ and C/EBP-α were effectively reversed by β-catenin siRNA (Fig. 4C). Additionally, TSN-induced inhibition of lipid accumulation was also reversed by β-catenin siRNA (Fig. 4D). Taken together, β-catenin is critically involved in the anti-adipogenic effects of TSN. Besides, we investigated the effects of TSN on MAPK signaling and the results showed that TSN significantly suppressed the phosphorylation of ERK1/2, JNK, and p-38 (Supporting Fig. 2).

### TSN represses gonadal white fat growth in HFD-fed C57 BL/C6 mice

**Figure 5:**
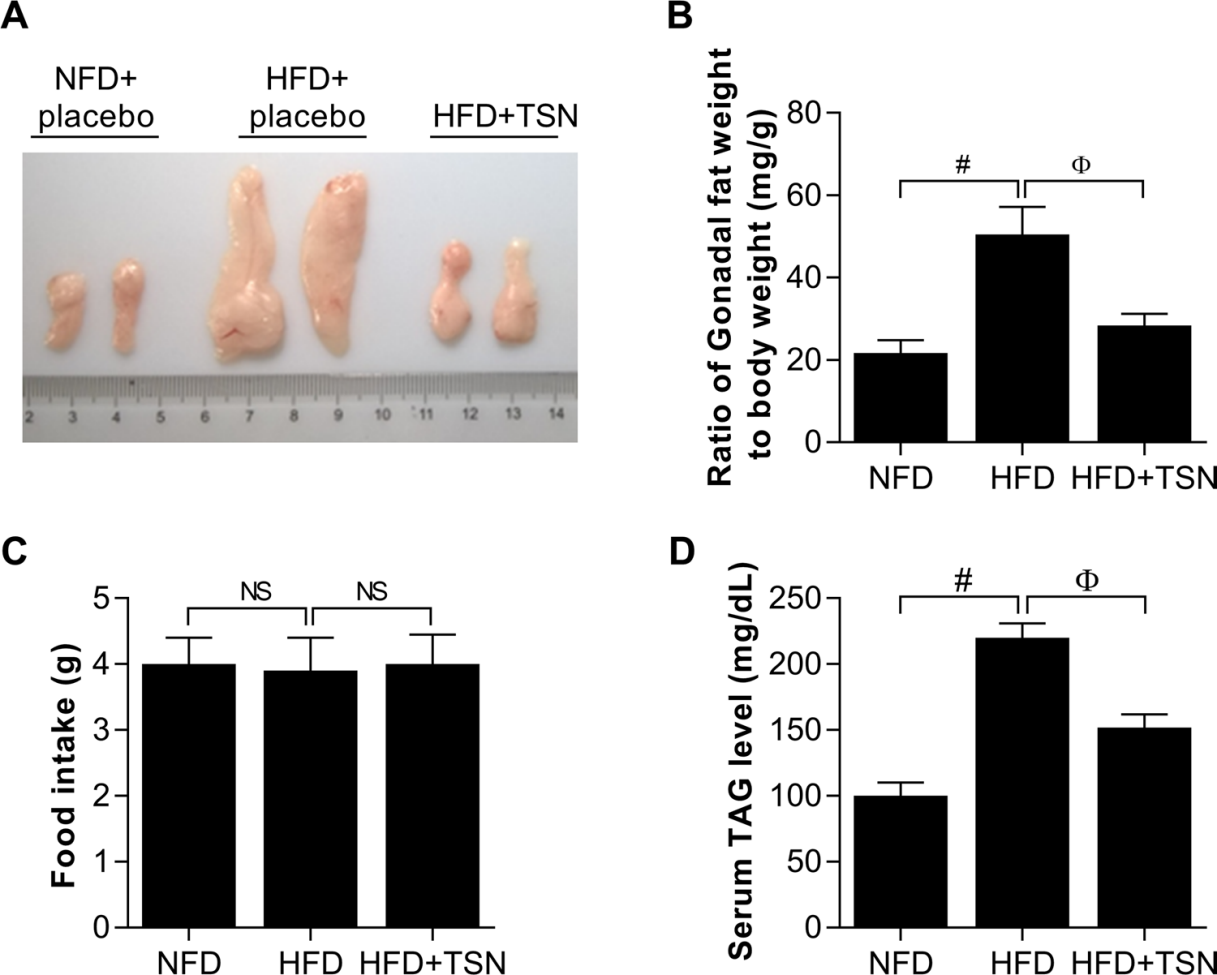
TSN represses gonadal fat growth in mice. Mice (C57 BL/6) were fed for one month and a half with NFD and HFD respectively. In the next period for one month, the mice were randomly grouped into NFD + placebo, HFD + placebo, HFD + TSN (0.1 mg/kg·day). (**A**) The size of gonadal fat from C57 BL/6, each grid represents 1 millimeter (1 mm). (**B**) The ratio of gonadal fat weight to body weight (mg/g). Values are mean ± SD of data from 8 replicates (n = 8); ^#^*P* < 0.001 (NFD + placebo vs HFD + placebo); ^Ф^*P* < 0.001 (HFD + placebo vs HFD + TSN). (**C**) and (**D**) Effects of TSN treatment on food intake (g) and serum TAG (mg/dL) levels of each group respectively. Values are mean ± SD of data from 8 replicates (n = 8); NS: no significance; ^#^*P* < 0.001 and ^Ф^*P* < 0.001.

To examine whether the inhibitory effect of TSN on lipid accumulation also occurs *in vivo*, we took the gonadal white fat tissue from mice as specimen for further analysis. After feeding with HFD for one month and a half, the mice were administered with TSN at 0.1 mg/kg·d or placebo for an additional month. As a result, TSN significantly suppressed the size and weight of gonadal white fat (Fig 5A,B). As compared with the model group, TSN significantly reduced the weight of gonadal white fat by 54.11%. However, TSN treatment failed to alter food intake in mice (Fig. 5C). Interestingly, TSN obviously downregulated serum TAG levels of HFD-induced mice by 30.91% (Fig. 5D), as compared with mice fed with HFD alone.

**Figure 6:**
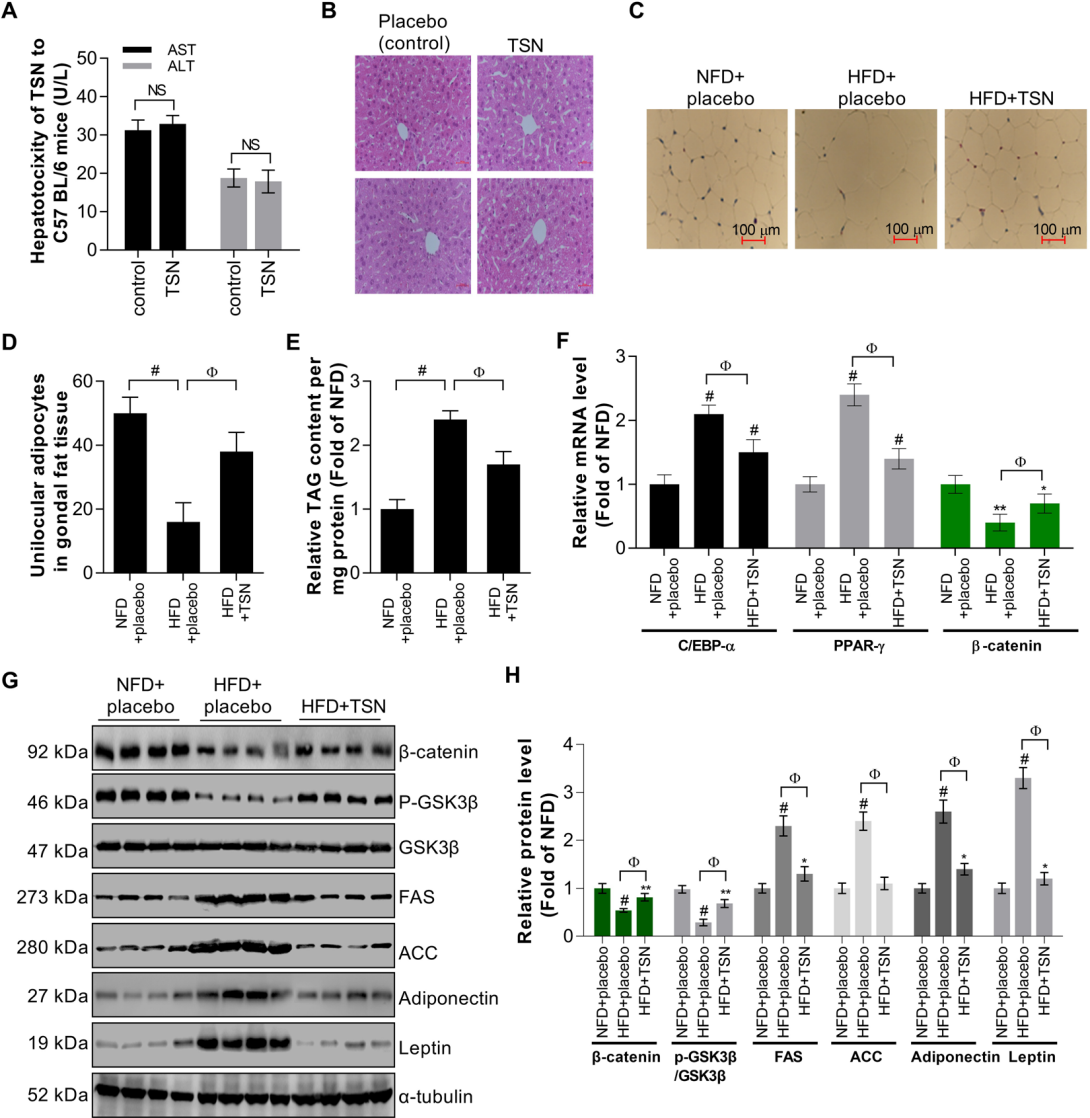
TSN suppresses lipid accumulation in gonadal fat tissue. (**A**) Examination of the effect of TSN on the levels of ALT and AST (U/L) in liver by ELISA. Values are mean ± SD of data from 8 replicates (n = 8); NS: no significance. (**B**) Effect of TSN on the tissue injury of liver (mice) detected by H & E staining. (**C**) Effects of TSN on unilocular adipocytes in gonadal fat tissue; number of unilocular adipocytes was calculated in a photo captured by a light microscope (10 × 40) and 20 photos for each group were chosen randomly for statistics. Scale bars: 100 μM. Values are mean ± SD of data and ^#^*P* < 0.001 (NFD + placebo vs HFD + placebo); ^Ф^*P* < 0.001 (HFD + placebo vs HFD + TSN). (**D**) Effects of TSN on TAG content of gonadal fat tissue in mice. Values are mean ± SD of data from 8 replicates (n = 8); ^#^*P* < 0.001 (NFD + placebo vs HFD + placebo); ^Ф^*P* < 0.001 (HFD + placebo vs HFD + TSN). (**E**) Effects of TSN treatment on the mRNA expression of C/EBP-α, PPAR-γ and β-catenin analyzed by real-time PCR. Values are mean ± SD of data from 8 replicates (n = 8); *P < 0.05, **P < 0.01 and ^#^*P* < 0.001 vs NFD + placebo; ^Ф^*P* < 0.001 (HFD + placebo vs HFD + TSN). (**G**,**H**) Effects of TSN on β-catenin, p-GSK3β, FAS, ACC, adiponectin, and leptin expression in gonadal fat tissue detected by western blot (**G**) and the corresponding semi-quantitative analysis was based on optical density assessed by image j software (**H**). Values are mean ± SD of data from 4 replicates (n = 4); **P < 0.01 and ^#^*P* < 0.001 vs NFD + placebo; ^Ф^*P* < 0.001 (HFD + placebo vs HFD + TSN).

Notably, we found that TSN, when used at a concentration of 0.1 mg/kg·d, failed to change the contents of serum ALT and AST (Fig. 6A). H&E staining assay also revealed that TSN treatment did not trigger significant hepatic injury in mice (Fig. 6B). Therefore, the dose for TSN used in this study is safe. Then, we observed fewer unilocular adipocytes in gonadal fat tissue occurred in mice after TSN treatment (38 ± 6 houses per microscope field) when compared with that of mice fed with HFD alone (16 ± 7 houses per microscope field) (Fig. 6C,D). TSN also suppressed the TAG content (the main component of lipid) of gonadal fat tissue by 29.17% (Fig. 6E), and reduced the mRNA expression of C/EBP-α and PPAR-γ of gonadal fat tissue by 28.57% and 41.66%, respectively. However, TSN upregulated β-catenin mRNA levels by 75% (Fig. 6F) as compared with that of mice fed with HFD alone. Finally, TSN prevented the downregulation of β-catenin in HFD-induced mice suppressing expression of FAS, ACC, adiponectin, and leptin (Fig. 6G,H). In sum, TSN may inhibit lipid accumulation in gonadal fat tissue through activating the Wnt/β-catenin signaling.

**Figure 7:**
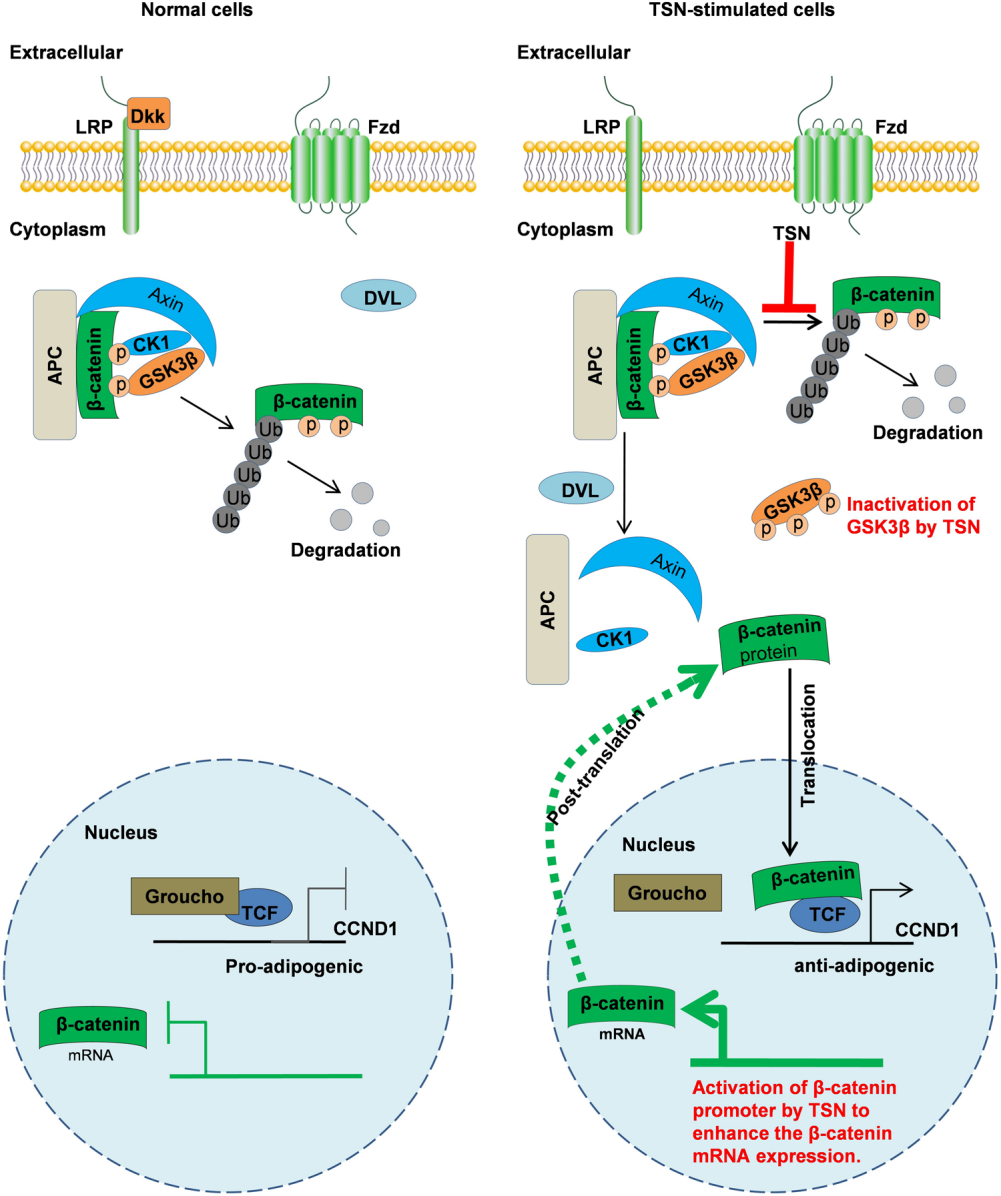
Schematic illustration of the effects of TSN on adipocyte cells differentiation. (**A**) In the absence of a Wnt signal, Dkk1 inhibits Wnt signaling by binding to co-receptors LRP. β-catenin is captured by a destruction complex that contains Axin and APC (adenomatous polyposis coli), which facilitates β-catenin phosphorylation by CKI and GSK3β. Then phosphorylated β-catenin is recognized by E3 ubiquitin ligase, which causes β-catenin to be degraded by the proteasome. (**B**) In TSN-stimulated cells, TSN enhances GSK3β phosphorylation, which results in β-catenin hypo-phosphorylation and its accumulation in cytoplasm. Additionally, TSN directly inhibits β-catenin ubiquitination, which also results in β-catenin accumulation in cytoplasm. Subsequently, β-catenin translocates into the nucleus and promotes the transcription of genes (such as CCD1) which inhibits adipogenic genes expression, such as C/EBP-α and PPAR-γ; FAS and ACC; adiponectin, and leptin. (**C**) On the other hand, TSN may activate the β-catenin promoter to enhance the β-catenin transcription, which results in overexpression of β-catenin mRNA.

Finally, we summarized this study as depicted in Fig. 7. In TSN-stimulated cells, TSN promotes GSK3β phosphorylation, which results in β-catenin hypo-phosphorylation and its accumulation in cytoplasm. Additionally, TSN might directly inhibit β-catenin degradation, which results in suppression of β-catenin degradation in cytoplasm. On the other hand, TSN may activate the β-catenin promoter to enhance the β-catenin transcription, which results in overexpression of β-catenin mRNA. Subsequently, β-catenin enters into the nucleus and upregulates the transcription of genes (such as CCD1) which inhibit adipogenic genes expression, such as C/EBP-α and PPAR-γ, FAS and ACC, adiponectin, and leptin. Consequently, TSN activates the WNT/β-catenin signaling by upregulating the expression of β-catenin at mRNA and protein levels.

## Discussion

As an active ingredient isolated from herbal plant, TSN has been originally identified as a selective presynaptic blocker and an effective antibotulismic agent^15–17^. However, increasing evidence demonstrates that TSN has additional activities and it is beneficial for the treatment of inflammatory diseases or cancer. In this study, we proposed for the first time that TSN is able to inhibit adipogenesis both *in vitro* and *in vivo* by activating the Wnt/β-catenin signaling.

The process of preadipocytes differentiation is regulated by a complex network of transcription factors. PPAR-γ and C/EBP-α are the master adipogenic transcription factors mainly found in adipose tissue^3^. The PPAR-γ is a ligand-activated transcription factor and it promotes the expression of several adipogenic and lipogenic genes in adipocytes^23^. It has been reported that ectopic expression of PPAR-γ alone is able to promote the entire adipogenic program in fibroblast cells, suggesting the key role of PPAR-γ in regulation of preadipocyte differentiation^3,24^. Also, the C/EBP-β and C/EBP-δ levels increase rapidly in hormonal stimulation and then synergistically trigger the expression of PPAR-γ and C/EBP-α, which cross-regulate each other through a positive feedback loop and result in the terminal differentiation^25^. In this study, we found that TSN inhibited PPAR-γ and C/EBP-α (not C/EBP-β and C/EBP-δ) mRNA and protein levels both *in vitro* and *in vivo*, which indicated that TSN inhibited adipogenesis by hindering the transcription factor cascade.

Next, the adipogenic transcription factors modulate the expression of adipocyte-specific genes, including FAS and ACC which are the essential lipogenic enzymes monitoring biosynthesis of fatty acids and triacylglycerols in the late stage of adipogenesis^3^. As showed in this study, TSN significantly decreased the expression of FAS and ACC, suggesting that TSN may negatively control lipid accumulation by modulating biosynthesis-related genes in differentiated 3T3-L1 adipocytes.

It is well known that the Wnt/β-catenin signaling reduces adipogenesis by blocking induction of PPAR-γ and C/EBP-α^7^. As a negative regulator of adipocyte differentiation, β-catenin can directly suppress the activity of PPAR-γ by means of the functional interaction between the TCF/LEF-binding domain of β-catenin and the catenin-binding domain of PPAR-γ^9,26^. The β-catenin can also decrease the activity of PPAR-γ and C/EBP-α by increasing CCND1 transcription^7,9,26^. In this study, the expressions of Wnt/β-catenin signaling proteins were upregulated by TSN in well-differentiated 3T3-L1 adipocytes as well as in HFD-fed mice. The β-catenin protein and mRNA levels were elevated by TSN through suppressing the degradation of β-catenin protein and enhancing the transcription of β-catenin mRNA, respectively. Moreover, TSN-induced upregulation of adipogenesis was reversed by β-catenin silencing. Hence, Wnt/β-catenin signaling plays critical role in the anti-adipogenic effect by TSN (Fig. 7). Future studies will be performed to examine whether TSN strengthens the interaction between β-catenin and PPAR-γ.

It has been reported that the Wnt/β-catenin pathway is activated when the secreted glycoprotein Wnt binds to a cell surface receptor, such as the frizzled receptor and LRP5/6 co-receptors^27^. The LRP6 inhibits adipogenesis and its deficiency spontaneously leads to adipogenic differentiation^7,8,28^. Similarly, DVL2 is the only activator in DVL family and regulates the disruption of Axin-GSK3β-APC complex; GSK3β is a repressor of the WNT/β-catenin pathway, and disruption of GSK3β stabilizes β-catenin and reduces adipocyte differentiation^7,8^. In the current study, we observed that TSN increased LRP6 and DVL2 expression levels in well-differentiated 3T3-L1 adipocytes, but inactivated GSK3β by enhancing its phosphorylation, which suggest that the stimulatory effect of TSN on β-catenin expression may be relevant to GSK3β. Notably, previous studies indicate that β-catenin gene transcription is critically dependent on transcriptional factors AP-1 and G3BP1^29,30^. Reactive oxygen species (ROS) may activate the AP-1 and G3BP1^31,32^, the second messenger Ca^2+^ can also stimulate the transcriptional activity of AP-1^33^. Interestingly, TSN increases Ca^2+^ influx in NG108-15 cells via L-type Ca^2+^ channels^34^, TSN can also trigger the generation of H_2_O_2_ in A549 cells^19^. Both ROS and Ca^2+^ are involved in the tumor-sensitizing effect of TSN on non-small-cell lung carcinoma^19^. Therefore, it seems reasonable to presume that TSN-induced upregulation of β-catenin is also regulated by Ca^2+^ and ROS signaling. Obviously, more experiments need to be done to clarify this presumption. Finally, the anti-adipogenesis of TSN imply that this chemical may also be useful in the treatment of fatty liver disease (FLD), because lipid accumulation in hepatocyte is a major characteristic of FLD. This concern will be addressed in further studies.

In conclusion, TSN inhibited adipocytes differentiation and lipid accumulation through activating Wnt/β-catenin signaling both *in vitro* and *in vivo* (Fig. 7). TSN might be employed as a natural anti-obesity agent for prevention and treatment of obesity.

## Electronic supplementary material

Data 1
