# Supplementary material for: Toosendanin inhibits adipogenesis by activating Wnt/β-catenin signaling
Source: Sci Rep. 2018 Mar 15;8:4626. doi: 10.1038/s41598-018-22873-x (PMC5854628; doi:10.1038/s41598-018-22873-x)

**Toosendanin inhibits adipogenesis by activating Wnt/β-catenin signaling**

Tian-xing Chen^1^, Xiao-ying Cheng^1^, Yun Wang^1^, and Wu Yin^1,2,3,*^

1.The State Key Lab of Pharmaceutical Biotechnology, College of life Sciences, Nanjing University, Nanjing, 210046, China.

2.Jiangsu Key Laboratory for Pharmacology and Safety Evaluation of Chinese Materia Medica, School of pharmacy, Nanjing University of Chinese Medicine, Nanjing, 210023, China.

3. Nanjing KMK Pharmaceutical Co., Nanjing, 210024, China

***Please address all correspondence to:**

Yin Wu Ph.D. 168# Xianlin Ave, Room No. A310-1, College of Life Sciences in Nanjing University (Xianlin Campus), Lab of Biochemical and Molecular Pharmacology (LBMP), State Key lab of Pharmaceutical Biotechnology (SKLPB), Nanjing, China,210046.

Email:wyin@nju.edu.cn, Phone: 0086-25-66099006.

**Supporting Figure 1. TSN activates the β-catenin promoter and inhibits the degradation of β-catenin promoter.** (**A**) TSN activates the β-catenin promoter. We constructed β-catenin promoter into pGL-3 basic plasmid. After 10 h transfection of the promoter plasmids, the 3T3-L1 adipocytes were incubated with TSN (50 nM) for another 12 h. The results showed that TSN significantly increased the luciferase activity of β-catenin promoter. (**B**) TSN inhibits the degradation of β-catenin. The 3T3-L1 adipocytes were incubated with cycloheximide (CHX, 2 μM) for half an hour, and then the cells were co-treated in presence or absence of TSN (50 nM) for another 3 h and 6 h.

**Supporting Figure 2. TSN inhibits the MAPK pathway in 3T3-L1 adipocytes.** Effects of TSN treatment on the phosphorylation of MAPKs including ERK, p38, and JNK analyzed by western blot. Tubulin-α was used as an internal control.

**Supporting Fig.1**


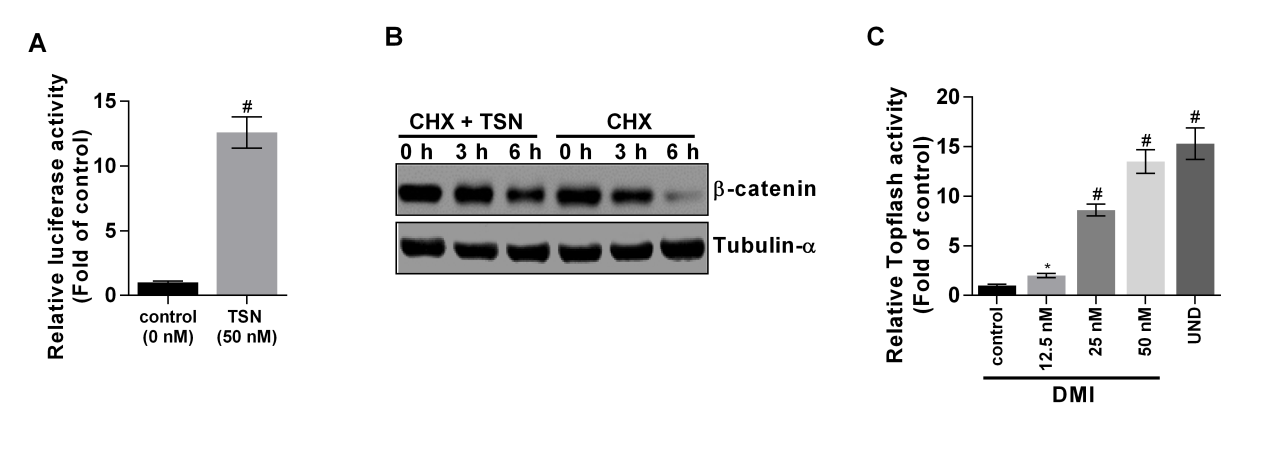


**Supporting Fig.2**


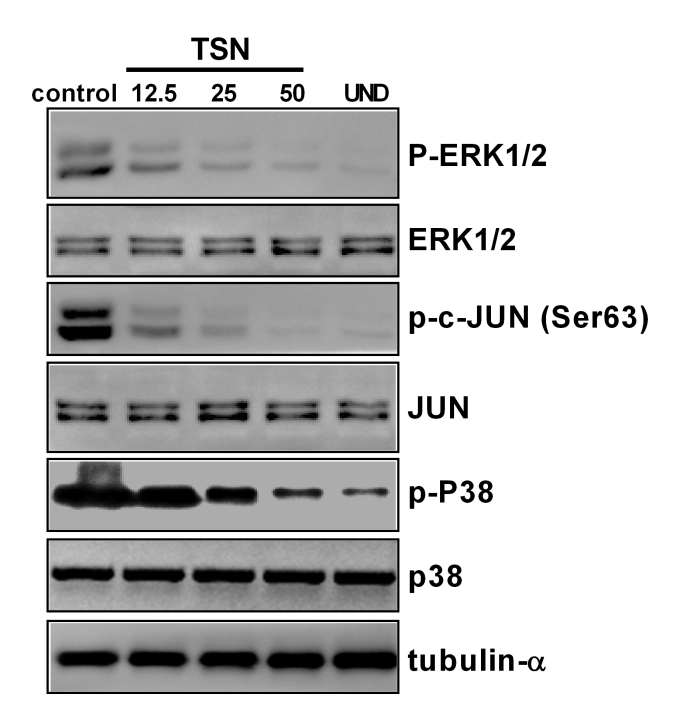

Supplement: Supplementary file 1 — Data 1 [file 41598_2018_22873_MOESM1_ESM.docx]
